# Mechanisms of ultrasonic de-agglomeration of oxides through *in-situ* high-speed observations and acoustic measurements

**DOI:** 10.1016/j.ultsonch.2021.105792

**Published:** 2021-10-15

**Authors:** Abhinav Priyadarshi, Mohammad Khavari, Tungky Subroto, Paul Prentice, Koulis Pericleous, Dmitry Eskin, John Durodola, Iakovos Tzanakis

**Affiliations:** a Faculty of Technology, Design and Environment, Oxford Brookes University, Oxford OX33 1HX, United Kingdom; b Brunel Centre for Advance Solidification Technology (BCAST), Brunel University London, Uxbridge UB8 3PH, United Kingdom; c Cavitation Laboratory, School of Engineering, University of Glasgow, Glasgow G12 8QQ, United Kingdom; d Computational Science and Engineering Group (CSEG), Department of Mathematics, University of Greenwich, London SE10 9LS, United Kingdom; e Tomsk State University, Tomsk 634050, Russia; f Department of Materials, University of Oxford, Oxford OX1 3PH, United Kingdom

**Keywords:** De-agglomeration, Dispersion, Oxides, Microbubble, High-speed imaging

## Abstract

Ultrasonic de-agglomeration and dispersion of oxides is important for a range of applications. In particular, in liquid metal, this is one of the ways to produce metal-matrix composites reinforced with micron and nano sized particles. The associated mechanism through which the de-agglomeration occurs has, however, only been conceptualized theoretically and not yet been validated with experimental observations. In this paper, the influence of ultrasonic cavitation on SiO_2_ and MgO agglomerates (commonly found in lightweight alloys as reinforcements) with individual particle sizes ranging between 0.5 and 10 μm was observed for the first time in-situ using high-speed imaging. Owing to the opacity of liquid metals, a de-agglomeration imaging experiment was carried out in de-ionised water with sequences captured at frame rates up to 50 kfps. In-situ observations were further accompanied by synchronised acoustic measurements using an advanced calibrated cavitometer, to reveal the effect of pressure amplitude arising from oscillating microbubbles on oxide de-agglomeration. Results showed that ultrasound-induced microbubble clusters pulsating chaotically, were predominantly responsible for the breakage and dispersion of oxide agglomerates. Such oscillating cavitation clusters were seen to capture the floating agglomerates resulting in their immediate disintegration. De-agglomeration of oxides occurred from both the surface and within the bulk of the aggregate. Microbubble clusters oscillating with associated emission frequencies at the subharmonic, 1st harmonic and low order ultra-harmonics of the driving frequency were deemed responsible for the breakage of the agglomerates.

## Highlights

• High-speed imaging captured de-agglomeration and dispersion of oxide aggregates.
• De-agglomeration occurred from both bulk and surface of the aggregate.
• Floating microbubble cluster drones produced fine suspension of loose agglomerates.
• Ultrasonic capillary effect promoted de-agglomeration of particles from within.
• Subharmonic and low order ultra-harmonic bubble emissions induced oxide breakage.

## Graphical abstract

**Figure:**
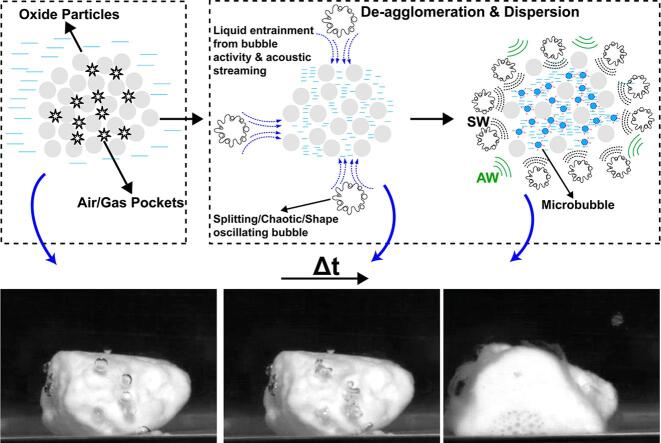

## 1 Introduction

Ultrasonic treatment (UST) of metallic melts favourably impacts material quality through grain refinement, particle mixing and, dispersion, cluster fragmentation, and degassing [1], [2]. This equally applies to liquid metal processing with added nanoparticles/powders or grain refining agents used to produce metal matrix composite materials [3], [4]. Incorporation of oxides such as alumina, silica and magnesia as reinforcements offers superior strength to the majority of aluminium alloy composites [5], [6], [7]. The use of such ceramic materials in Al matrix composites is considered suitable as it acts as particulate reinforcement that offers high hardness, wear resistance, compressive strength, and thermal stability [8]. Other than in composite materials, the use of oxides like MgO have been found to be useful in other applications such as waste water treatment [9], antacid for heartburn and dyspepsia [10] and also as an anticaking agent in food additives [11]. In all these applications, there is a challenge of agglomeration that needs to be addressed. Nevertheless, there are issues with the introduction of such particles in melts and more specifically to liquid aluminium [12]. Owing to large surface tension, poor wettability, oxidation and hydrogen adsorption, particles added externally, or indigenous oxides, often form agglomerates with absorbed hydrogen on their surface [1]. Thus, these agglomerated particles cannot be wetted by the surrounding melt and are particularly difficult to disperse and distribute uniformly within the liquid metal matrix. UST has been found to assist in both distribution and dispersion of particles added or formed within the melt through the process known as ultrasonic de-agglomeration [13], [14]. The ultrasound-induced acoustic cavitation and related streaming effects, both play an important role in de-agglomeration and subsequent dispersion of particles in the liquid. Note that this is important not only for the metal-matrix composites but also for “conventional” grain refinement that is based on the distribution of secondary phases or activated substrates, which is facilitated by de-agglomeration/dispersion. Beyond liquid metal processing, de-agglomeration using ultrasonic effects can be important in other applications ranging from pharmaceuticals to food industry [15].

It is hypothesized that the increased wettability of the particles under cavitation conditions allows the molten metal to reach their surface and subsequently penetrate the agglomerate through capillaries [16]. This has been resolved experimentally using synchrotron studies where the sono-capillary mechanism in molten aluminium was captured, showing that the high-speed liquid jets from the collapsing bubbles lead to the de-agglomeration of oxide particles trapped within a groove during the solidification process [17]. However, the role of ultrasonic cavitation on the particle de-agglomeration and the underlying mechanisms have not yet been clearly understood and have only been qualitatively described. For example, Kudryashova and Vorozhtsov [13] demonstrated analytically that the cavitation-induced microscopic bubbles pulsate and collapse near the agglomerate, developing overpressure that pushes the melt into the narrow pores of an agglomerate by overcoming the capillary pressure threshold. The liquid thus enters the capillary channels and pores of the agglomerates. The infusion of liquid metal into the agglomerated particles considerably alters the properties of the agglomerates and expedites the breakup of the particle cluster (de-agglomeration) through viscous and shear forces caused by the cavitating bubbles and propagating ultrasonic waves. The high intensity ultrasonic waves produce numerous microscopic cavitating bubbles that are distributed within the liquid volume by the generated acoustic and secondary flows. Moreover, as the bubbles favourably nucleate at gas pockets and interfaces, pores within the aggregate and individual particles act as ideal sites for cavitation nucleation. Eskin and Eskin [1] postulated the de-agglomeration mechanism in a slightly more comprehensive manner. The mechanism was described as follows: The formation of microbubbles initially occurs at the particle/liquid interfaces (gas pockets). As the bubbles tend to pulsate vigorously under the influence of ultrasound, this causes the loosening of agglomerates from within. The resulting pressure and momentum impulses as bubbles collapse break the agglomerates apart. The generated localised pressure, which reaches up to 500 MPa [18], [19] is sufficient to overcome the capillary and adhesive bonds between the particles (∼1 MPa) within the agglomerate [20]. The acoustically induced flows further distribute the particles within the treated volume. Up to now, most experiments on ultrasonic de-agglomeration in metallic melts have been performed in ex-situ and post-mortem conditions and the associated effects were interpreted based on microscopic examination of the formed particle clusters in the solidified product. For example, Eskin et al. [21] experimentally observed the partial de-agglomeration of TiB_2_ clusters by remelting a commercial Al-3%Ti-1%B master alloy and by ultrasonically treating the alloy melt using a 5-kW transducer operating at a frequency of 18 kHz. It was found that the cavitation treatment of an AlTiB master alloy resulted in the release of individual TiB_2_ particles of sizes between 1 and 3 μm from the agglomerates and they further became available as active substrates to promote heterogeneous nucleation of new aluminium grains. However, in-situ studies to understand the de-agglomeration mechanism are scarce, owing to the liquid metal opacity that makes real-time observations difficult. Water has long been recognised as a suitable liquid analogue system for studying the effects of intermetallic fragmentation and de-agglomeration [1], [22], [23], [24], [25], [26], [27], [28]. Eskin et al. [25] recently showed through in-situ high-speed observations that cavitation assisted de-agglomeration of MgO aggregates in water was initiated from their outer surface and not from within. The de-agglomeration process was thought to resemble the ‘chipping-off’ of individual constituent particles from the surface rather than occurring within the bulk. Nevertheless, there is still a deficit in the literature on studying the process of de-agglomeration of particles within ultrasonically treated liquid and analysing the effect of cavitation bubbles and streaming on particle dispersion and distribution.

The present work focuses on understanding through real-time high-speed imaging, of the overall de-agglomeration process of silica (SiO_2_) and magnesia (MgO) agglomerates with individual particle sizes up to 10 μm exposed to cavitation action in water, under the influence of ultrasound. In addition to being model oxide agglomerates, silica and magnesia are widely used in cosmetics, food, and nutritional supplements; hence, their de-agglomeration behaviour has broader practical significance. The underlying mechanism of group and individual agglomerates has been explained in terms of ultrasonic cavitation and induced acoustic streaming effects. The de-agglomeration process has also been further characterised in terms of the frequency components observed within the cavitation emission signals.

## 2 Materials and methods

### 2.1 In-situ high-speed imaging

**Fig. 1:**
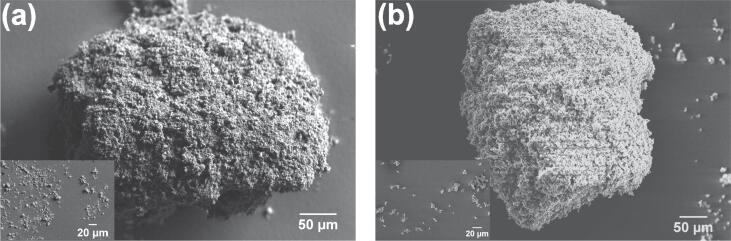
Microstructural images showing the typical surface morphology of (a) SiO_2_, and (b) MgO agglomerate with individual particle size up to 10 μm depicted as an inset.

**Fig. 2:**
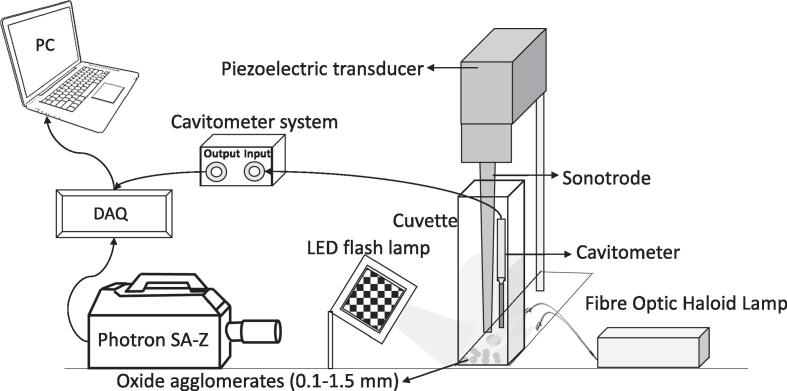
Schematic representation of the experimental setup used for obtaining synchronised acoustic pressure measurements and in-situ imaging of ultrasonic de-agglomeration.

High purity SiO_2_ (99.999%) and MgO (99.998%) were obtained from Fisher Scientific, UK having particle density of 2.65 and 3.58 g/cm^3^, respectively. Agglomerates of these oxides with particle sizes in the range of 0.5–10 μm were used for ultrasonic de-agglomeration experiments in deionised water. Fig. 1 shows the microstructural images of silica and magnesia aggregates and individual oxide particles (as inset) obtained using scanning electron microscopy (SEM) prior to exposure to ultrasound. The in-situ high-speed observations were performed in a transparent cuvette of dimensions, L: 12 mm; W: 12 mm; H: 44 mm, using a 200 W piezoelectric transducer (UP200S, Hielscher Ultrasonics GmbH, Germany) operating at 24 kHz with a coupled titanium sonotrode (tip diameter Ø = 3 mm). Detailed specification of this ultrasonic device can be found elsewhere [29]. The liquid height in the cuvette was maintained at 20 ± 2 mm and the sonotrode was submerged 15 ± 1 mm below the liquid surface. The tip-vibration amplitude of the sonotrode was chosen to be 210 μm peak to peak (for developed cavitation). The experimental test rig designed for the experiments is depicted schematically in Fig. 2. The high-speed imaging of the de-agglomeration effect was undertaken using Fastcam SA-Z 2100 K (Photron, UK) and the cavitation activity was recorded at 20,000–50,000 frames per second (fps), with a shutter speed of 18.39 μs through a Navitar 12 × zoom lens (0.5 × 0.009–0.051NA 1–50012). At 50 kfps, imaging was obtained over 896 × 448 pixels with a resolution of 11.2 μm/pixel. High intensity light illumination was provided by a GS VITEC Multi LED flash lamp and a Fibre Optic Haloid lamp from front and rear end of the focussing plane, respectively. This illumination allowed for observing bubbles in both the transient and stable oscillation modes. The main purpose of this imaging was to capture the de-agglomeration sequence of the oxide agglomerates under the influence of ultrasonic cavitation and acoustic streaming effects.

### 2.2 Acoustic detection

The acoustic emissions ware detected using a bespoke low-frequency (in the range of kHz) calibrated cavitometer mounted 3 ± 0.2 and 0.8 ± 0.1 mm away from the centre of the horn tip in transverse and longitudinal direction, respectively (Fig. 2). This bespoke cavitometer comprised an in-built tungsten waveguide (Ø = 4 mm) connected to a piezoelectric sensor that converted the acquired mechanical vibrations into a voltage signal. The cavitometer had a spatial resolution of 40 mm and was calibrated in the National Physical Laboratory (NPL, UK) over a frequency range of 8–400 kHz with a sensitivity function as described in [30]. Spatial resolution of any sensor defines its ability to resolve the cavitation activity occurring within the ultrasonically treated volume. The significance of spatial resolution is that it allows for the characterisation of the whole cavitating environment with greater understanding of the treatment volumes, which can help in scaling up the sonication systems. The treatment volumes used for de-agglomeration studies in this paper were very small with a cuvette length of 12 mm, therefore the spatial resolution of 40 mm was adequate to acoustically characterise the overall treatment volume. Detailed information regarding the performance, design and specification of this cavitometer can be found elsewhere [31]. The bandwidth of the cavitometer extended up to 10 MHz [32], which was sufficient to enable monitoring of the broadband acoustic emissions resulting from the cavitation activity. The calibration sensitivity of the cavitometer at the driving frequency of the ultrasound was around −279 dB re 1 V/µPa. For the experiments described below, the cavitometer was strategically synchronised with the camera and the ultrasonic device using a data acquisition (DAQ) system, at a sampling rate of 20 × 10^6^ samples per second. The acoustic measurements were acquired from the onset of cavitation activity extending for up to tens of milliseconds to account for the de-agglomeration of particles confined within the imaging field of view. The analysis of the raw data (voltage–time) obtained from the cavitometer was performed using an in-house developed MATLAB code by applying a Fast Fourier Transformation (FFT) to the captured signal as described elsewhere [30], [33], [34], to appropriately apply the sensitivity values of the sensor. The output of the acoustic emissions was displayed in the pressure–time domain after subtracting the background noise. The spectrum was obtained corresponding to the FFT of ∼ 50 ms signal with a resolution of 0.02 kHz (or 20 Hz). The acoustic measurements were then related to the observed de-agglomeration sequence obtained using high-speed imaging.

## 3 Results and discussion

### 3.1 High-speed observations of ultrasonic de-agglomeration

#### 3.1.1 Silica (SiO_2_) aggregates

**Fig. 3:**
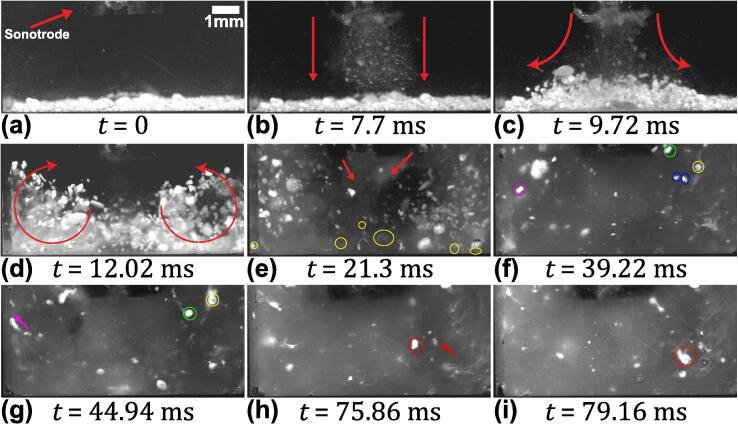
Images of SiO_2_ de-agglomeration caused by the cavitation bubble clusters and induced acoustic streaming flow recorded at 50 kfps with sonotrode-tip operating at 210 μm peak-to-peak amplitude. Note that the resolution is not high enough to resolve individual particles that are seen as “mist” in (e). See Supplementary video 1.

Fig. 3 (see Supplementary video 1) represents a high-speed image sequence of SiO_2_ aggregates subjected to de-agglomeration captured from the onset of cavitation. Time, t = 0 ms shows the number of agglomerates of varying sizes positioned ∼ 3.5 mm below the sonotrode tip (Fig. 3a). Once the ultrasound was turned on, numerous microbubbles were formed and propagated from the horn tip, forming a cavitation stream extending down to the agglomerate bed as shown in Fig. 3b. As the cavitation stream interacted with the agglomerates, those that were in the path of the bubble flow were set in motion to form recirculation vortices apparent on either side of the jet, as shown in Fig. 3(c-d). As the agglomerates were uplifted from the base, some of them moved towards the ultrasonic source and de-agglomerated instantly upon interacting with the strong cavitation cloud formed below the tip, generating a fine suspension in this region (Fig. 3e). In addition to the formation of primary cavitation bubbles, there were also some small microbubble (μB) clusters generated from the collapse of bubbles in the large cavitation cloud. These clusters underwent continuous splitting and coalescence as shown in Fig. 3f (marked with yellow contours). We shall call these bubble clusters, arising due to the Bjerknes forces created by the acoustic waves, ‘ultrasonic splitting microbubbles’. They pulsate chaotically undergoing continuous shape oscillations and move around in the cavitation field as previously reported by Kim et al. [35]. A surprising feature that has not, to the best of our knowledge, been previously reported was that these μB clusters attached themselves to the flowing agglomerates, triggering their immediate breakup. Two agglomerates in Fig. 3f (encircled in blue) flowed towards the bubble cluster (encircled in yellow). As the agglomerates reached the vicinity of the cluster, they were caught up in a pulsating μB cluster, causing them to de-agglomerate completely at t = 44.94 ms. Also, the aggregates (encircled in pink) de-agglomerated while in motion leaving a trail of very fine particle, like a comet, as shown in Fig. 3g (arrowed pink). Similarly, another moving agglomerate as seen in Fig. 3h (encircled in red) got captured by the μB cluster (marked with red arrow) and de-agglomerated almost instantly as shown in Fig. 3i. It is also interesting to note that in most of the cases the visual impression of de-agglomeration is the increase in size of the aggregates as seen in Fig. 3 (h-i). Eventually, owing to the large number of agglomerates present within the liquid volume, the visibility was impeded following their de-agglomeration, making the field of view too obscured for further meaningful observations. In the next experimental run, fewer agglomerated particles were deployed to overcome this visual constraint.

**Fig. 4:**
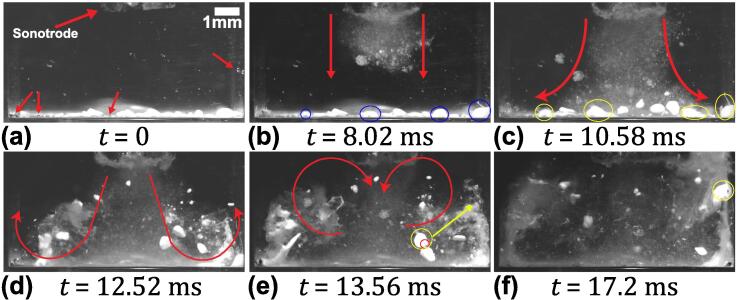
Sequence of images showing de-agglomeration and dispersion of fewer oxide aggregates under the influence of ultrasound captured at 50 kfps with a sonotrode amplitude of 210 μm peak-to-peak amplitude. See Supplementary video 2.

Fig. 4 (see Supplementary video 2) depicts representative frames from a high-speed sequence of SiO_2_ de-agglomeration under the same ultrasonic processing conditions as above, but with fewer aggregates this time. Fig. 4a shows the snapshot of agglomerates at the bottom of a water-filled cuvette, just before the onset of cavitation. At t = 0 ms, a number of gas bubbles (marked with red arrow) were present at the bottom, sidewalls and the corners of the cuvette. As the oxide agglomerates are highly porous in nature, numerous gas bubbles are expected to be present on their surface and the pores then act as preferred nucleation sites. After the transducer was switched on, the sonotrode started to vibrate causing the cavitation to initiate, thereby releasing numerous μB and μB clusters generated from strong bubble cloud collapses (Fig. 4b). With the onset of cavitation, the gas bubbles also started to undergo vigorous volumetric oscillations and moved towards some of the nearby agglomerates (encircled in blue). These bubbles then tended to oscillate on the surface of the aggregates shaking them loose from the outside, initiating the de-agglomeration process. This activity occurred even before the cavitation bubble stream reached the base of the cuvette. As soon as the microbubble stream reached the base, the agglomerates were pushed aside and the loosely bonded particles of the agglomerates were dispersed into the bulk liquid (Fig. 4c). Subsequently, the recirculation vortices formed on either side of main cavitation stream caused the agglomerates to lift-off from the base and drove them towards the ultrasonic horn-tip, thus producing further de-agglomeration of aggregates from the surface through induced acoustic streaming (Fig. 4d). Fig. 4e shows the instant when one of the floating agglomerates (encircled in red) with size approximately 576 μm was captured by a μB cluster (encircled in yellow) around 186 μm in size. The pulsating μB attached to and quickly eroded the agglomerate, and dispersed the individual particles into the bulk liquid volume while in motion as indicated with an arrow (Fig. 4f). Interestingly, even though these vigorously μB clusters are almost 3 times smaller in size than the oxide agglomerates, they can effectively disintegrate the aggregates into fine powders in just a few milliseconds. The actual mechanism through which the attached pulsating μB clusters caused de-agglomeration of the oxide aggregates could not be resolved in this experiment. Therefore, we deliberately captured the de-agglomeration process of a single SiO_2_ agglomerate.

**Fig. 5:**
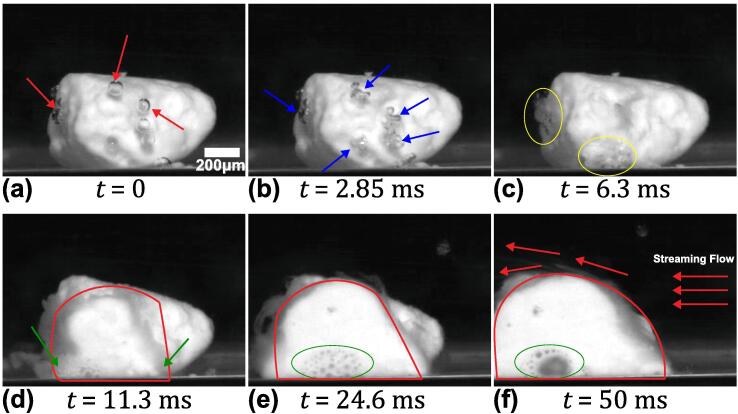
Sequence of zoomed view of de-agglomeration of a single SiO_2_ agglomerate under cavitation action captured at 20 kfps with sonotrode operating at 210 μm peak-to-peak amplitude. See Supplementary video 3.

Fig. 5 (see Supplementary video 3) shows the zoomed view of the de-agglomeration sequence of a single SiO_2_ agglomerate when exposed to ultrasonic waves. Fig. 5a, at t = 0 ms, shows a non-spherical agglomerate of 1 mm approximate size positioned at the corner of the cuvette and about ∼ 10 mm away for the horn tip in the longitudinal direction, with some gas bubbles attached to its surface (marked with red arrows). The position of the SiO_2_ aggregate for this particular observation was judiciously chosen to allow for capturing the de-agglomeration sequence in more detail, overcoming the visual constraints arising from the obscured field of view. When the ultrasound was switched on, the propagating acoustic waves (AW) in the liquid triggered these gas bubbles to volumetrically oscillate causing them to split and coalesce continuously as shown by blue arrows, Fig. 5b. As these chaotically oscillating μB clusters constantly imploded and rebounded on the surface, they produced erosion from the surface of the agglomerate possibly through the chipping mechanism most likely caused by the emitted micro-jets discussed elsewhere [25], [36]. Subsequently, the pulsating μB cluster tended to coalesce with nearby clusters causing them to collapse and rebound even more violently close to the agglomerate surface (marked in yellow contour) as shown in Fig. 5c. Stepišnik et al. [37] also reported that the dissolution of a surfactant could be caused primarily by the by cavitation bubbles imploding in its vicinity releasing multiple microjets leading to material erosion from the surface. These chaotically oscillating but stable μB clusters triggered, what resembles a landslide, where loose particles started to detach from the agglomerate and slide from the surface to create a suspension cloud (indicated with green arrow) as shown in Fig. 5d. Simultaneously, the agglomerate also became slightly distended from the front as the cluster oscillation continued and the bubbles seemed to penetrate inside it as shown by the green encirclement in Fig. 5(e-f). With increase in the number of acoustic cycles, the size of the μB cluster increased gradually causing the agglomerate to inflate even further (Fig. 5e). The induced acoustic flow from the ultrasonic source (indicated with red arrow) then pushed the agglomerate in the flow direction and dispersed the de-agglomerated particles into the liquid (Fig. 5f).

#### 3.1.2 Magnesia (MgO) aggregates

**Fig. 6:**
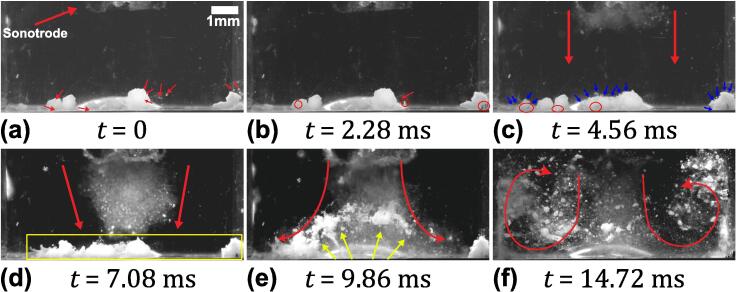
Sequence of images showing de-agglomeration and dispersion of MgO aggregates under the influence of ultrasound captured at 50 kfps with sonotrode amplitude of 210 μm peak-to-peak amplitude. See Supplementary video 4.

Fig. 6 (see Supplementary video 4) shows the frame-by-frame capture sequence of cavitation-induced de-agglomeration for MgO agglomerates. The first frame, t = 0 ms, shows the agglomerates located almost 3.5 mm below the horn tip, Fig. 6a. The frame also shows the presence of gas bubbles located on the bottom, sidewalls and on the surface of the agglomerate (indicated with red arrow). With the onset of cavitation, the gas bubbles began to pulsate, coalesced with other oscillating bubbles (encircled in red), and moved towards the nearby agglomerate (Fig. 6b) as was also observed in case of SiO_2_ agglomerates. Continued pulsation of these bubbles loosened the surface of the aggregate and initiated de-agglomeration through chipping-off of individual particles from the outer surface as indicated by blue arrows (Fig. 6c). The oscillation of the bubble clusters became even more vigorous as they coalesced with other such clusters surrounding the agglomerate surface and subsequently intensified the de-agglomeration rate, well before the cavitation bubble stream reached the agglomerate (Fig. 6d). Upon interaction with the cavitation cloud stream, the loosely bonded particles dispersed, moving into the bulk liquid (indicated with yellow arrows) as shown in Fig. 6e. The recirculation vortices formed on either side of the central cavitation stream further caused the broken-off agglomerates to flow into the cavitation zone (beneath the horn tip) for further de-agglomeration as previously seen in the case of SiO_2_ (Fig. 6f). To understand the role of these bubble cluster oscillations on de-agglomeration, a zoomed imaging of a single agglomerate was performed.

**Fig. 7:**
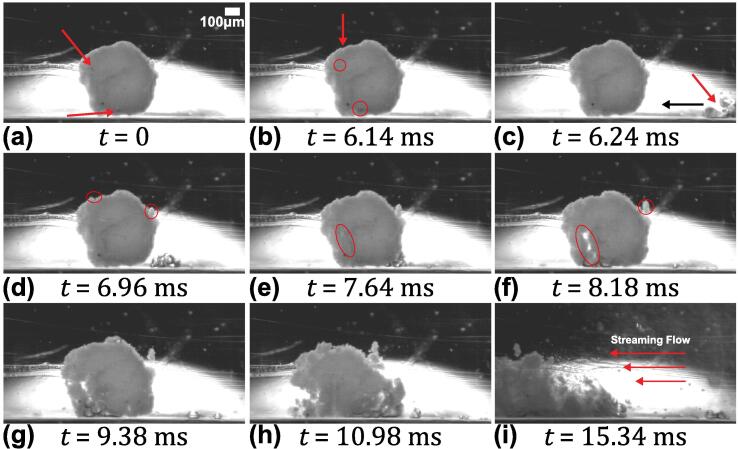
Sequence of zoomed view of de-agglomeration of a single MgO agglomerate under cavitation action captured at 20 kfps with sonotrode operating at 210 μm peak-to-peak amplitude. See Supplementary video 5.

Fig. 7 (see Supplementary video 5) displays the sequences of images showing the de-agglomeration process of a single MgO agglomerate caused by the oscillating bubble cluster under the influence of ultrasound. The agglomerate was positioned ∼ 3.5 mm away from the sonotrode tip and closer to the centre of the cuvette. Fig. 7a shows the agglomerate with visible gas bubbles approximately 20 μm in size on its surface (indicated with arrow). These bubbles started to pulsate stably as soon as the ultrasound was turned on (Fig. 7b). As seen in Fig. 7b, the tiny gas bubbles coalesced together to form a bubble cluster. A cluster of oscillating bubbles was also seen to move towards the agglomerate (Fig. 7c). These oscillating bubbles caused the agglomerate to become loosened from both inside and outside and further initiated surface rupture at the periphery (encircled in red) as shown in Fig. 7d. It is possible that the bubble clusters oscillating close to the agglomerate excited the tiny bubbles present within the pores and pushed the particles apart resulting in agglomerate breakage. Another possibility is that the AW generated sufficient pressure that pushed the surrounding liquid inside the pores of the agglomerate inducing breakup through sono-capillary effect [13], [14], [16], [17]. Once the cluster contacted the agglomerate, its oscillation amplitude further increased, accompanied with the appearance of subharmonics in the frequency spectrum (as seen later in section 3.2 and Fig. 9(a2)). The subharmonic emissions predominantly occurred as the large bubble clouds formed beneath the horn tip imploded after one or more cycles, emitting high energy shock waves and liquid jets [26], [38], a phenomenon sometimes termed as acoustic supercavitation [38]. In addition to the strong cloud collapses, as microbubble clusters approached the MgO aggregate, the amplitude of its oscillation increased and the cluster underwent repetitive violent collapses from time t = 9.40 ms to t = 10.98 ms. The frequency of collapse was as low as 1/8th of the fundamental between time t = 9.70 ms to t = 9.98 ms (see Supplementary video 5), expediting the de-agglomeration through rupturing the agglomerate as shown in Fig. 7(g-h). The de-agglomerated particles were then dispersed in the bulk liquid by the incoming acoustic streaming (Fig. 7i).

As observed in Fig. 3, Fig. 4, Fig. 5, Fig. 6, Fig. 7, it is evident that the de-agglomeration of oxides primarily occurs through cavitation bubbles that are formed within the ultrasonically treated liquid. The stable bubbles (splitting and shape oscillating microbubble clusters) initiate the de-agglomeration of aggregates through the chipping mechanism as described in Fig. 5b and Fig. 6c and earlier reported by Eskin et al. [25]. These stably oscillating bubbles then loose their shape and their oscillations becomes extremely violent with chaotic pulsations and continuous splitting thereby releasing multiple daughter bubbles, with the resulting liquid microjets with velocities of the order of 1 m/s [35] making the surface of the agglomerate inflated by the penetration of microbubbles and the induced sono-capillary effect as discussed later in Section 3.3 . The oxide particles thus becomes loose from both the surface and the bulk of the aggregate increasing the rate of de-agglomeration. However, the de-agglomeration caused by the transiently cavitating bubbles is more aggressive with powerful emitted shock waves generated from the subharmonic cloud collapses occurring at the tip of the horn. In this case, the de-agglomeration occurs instantly through rupture of the aggregate forming a ‘mist’ like region as seen in Fig. 3e when the agglomerates recirculate back towards the ultrasonic source due to the secondary vortex flow. Therefore, the de-agglomeration process is enhanced owing to the contribution from both the cavitation bubbles and the induced acoustic streaming. In the next section, the effect of pressure amplitude and cavitation emissions arising from oscillating microbubbles on oxide de-agglomeration will be presented and linked to the high-speed camera observations.

### 3.2 Acoustic pressure measurement

**Fig. 8:**
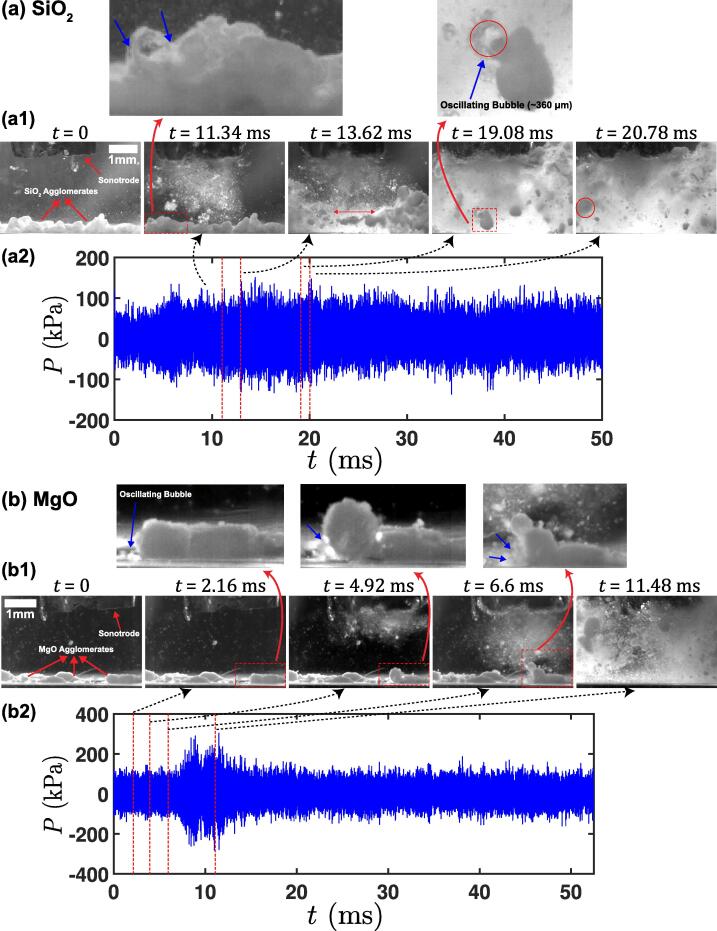
Synchronised high-speed image snapshot and in-situ acoustic pressure emission recorded using the advanced calibrated cavitometer showing de-agglomeration of (a) SiO_2_, and (b) MgO aggregates.

After detailed observations demonstrating some aspects of the mechanisms of de-agglomeration, we now delve deeper into the acoustic characterisation of the ultrasonic cavitating field that promotes de-agglomeration. Synchronised pressure measurement coupled with in-situ imaging of ultrasonic de-agglomeration were performed using a cavitometer sensor positioned close to the tip of the sonotrode as mentioned in Section 2.2. It should be noted that the acoustic pressure values reported here were measured with reference to barometric pressure since the cavitometer was calibrated in water at ambient pressure conditions as previously reported in [30]. Fig. 8a (see Supplementary video 6) displays the synchronised high-speed images (Fig. 8(a1)) and acoustic pressure–time profile (Fig. 8(a2)) for the de-agglomeration sequence of SiO_2_ agglomerates. The root mean square (RMS) and maximum pressures recorded were found to be approximately 32 kPa and 150 kPa, respectively. The de-agglomeration of SiO_2_ aggregates started before the bubble cloud stream interacted with them (t = 11.34 ms). With the increase in acoustic pressure i.e. after 5 ms, a large agglomerate (marked with double sided red arrow) was seen to break apart at t = 13.62 ms. As the pressure stabilised within the cavitating field, the oscillating μB cluster attached itself to one of the flowing agglomerates (indicated with red arrow) at t = 19.08 ms, triggered its breakup (t = 20.78 ms). Fig. 8b (see Supplementary video 7) shows the de-agglomeration imaging (Fig. 8(b1) and acoustic pressure variation (Fig. 8(b2)) across the cavitation field for MgO oxides. The RMS and maximum pressures generated within the cavitation field were found to be close to 46 kPa and 300 kPa, respectively. The slight increase in acoustic pressure values can be attributed to a smaller bubble concentration resulting in lower shielding [33], owing to the presence of fewer agglomerates compared to SiO_2_. With the introduction of ultrasonic waves to the liquid, gas bubbles with diameters in the range of 50–60 μm in diameter located near the agglomerates started to pulsate and moved closer to the oxides (t = 2.16 ms). As the cavitation activity intensified, the bubbles expanded and underwent vigorous pulsations close to the agglomerate (t = 4.92 ms) leading to its partial erosion from the surface even before the cavitation stream reached the agglomerate surface (t = 6.60 ms) as indicated with a blue arrow similar to observations made in case of SiO_2_. The pressure gradually escalated with increased cavitation activity up to 8.28 ms, reaching almost 300 kPa. The increased acoustic pressure thus expedited the de-agglomeration of loosened agglomerates and dispersed the oxide particles in the bulk liquid with the help of induced streaming flow (t = 11.48 ms). It is interesting to note from the pressure–time profile in Fig. 8 (a and b) that the cavitation output signal first increased after the onset of cavitation, reached a maximum value and then decreased and stabilized with the sonication time. This trend shows that the acoustic pressure generated by ultrasound increased until the cavitation cloud attached to the horn tip attained the maximum size and collapsed, thereby releasing high-energy shock waves and high-speed liquid jets as the cavitation activity increased [39]. The further decrease and stabilisation of the pressure was a result of non-collapsing bubble deflations [38] and collapses occurring after one or more acoustic pressure cycles.

**Fig. 9:**
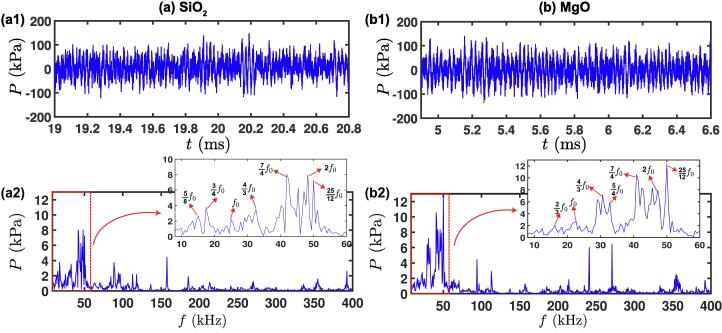
Acoustic pressure spectrum obtained after FFT of isolated pressure–time profiles for de-agglomeration of (a) SiO_2_, and (b) MgO.

To further understand the frequency content of the acoustic emissions from these μB clusters and its relationship to the de-agglomeration of SiO_2_ and MgO aggregates as observed in Fig. 8a (t = 19.08 to 20.78 ms) and Fig. 8b (t = 4.90 to 6.60 ms), respectively, the pressure–time profiles for both sequences were isolated and a fast Fourier transform (FFT) was applied to obtain the pressure–frequency spectrum.

Fig. 9 shows the acoustic pressure emissions in both time (see a1 and b1) and frequency (see a2 and b2) domains emitted from majority of the oscillating μB clusters responsible for inducing de-agglomeration. To obtain the acoustic spectrum, pressures profiles were carefully selected and isolated for FFT analysis based on the most prominent de-agglomeration events captured by the synchronised camera observations in Fig. 8a and 8b. Specifically, the time band selected for the analysis in the case of SiO_2_ i.e. from t = 19.08 ms to t = 20.78 ms and MgO, i.e. from t = 4.90 ms to t = 6.60 ms, was the period when the oscillating microbubble attached to the nearby aggregate and initiated the de-agglomeration process. The time span (acoustic cycles) was kept the same for comparison purposes. As can be seen, the pressure-frequency spectrum obtained from SiO_2_ de-agglomeration (Fig. 9 (a2)) shows multiple high frequency peaks extending up to 400 kHz, accounting for the non-linear and complex dynamics of microbubbles with prominent peaks at the low frequency region as also seen from camera observations between t = 19.08 to 20.78 ms. Zooming on the low frequency spectrum up to 60 kHz showed the fundamental (f_0_), 1st harmonic (2f_0_) and ultra-harmonics with 7f_0_/4, 25f_0_/12 as prominent higher order peaks with pressure magnitude of approximately 8 kPa in addition to lower order ultraharmonic (4f_0_/3) and subharmonic peaks of 5f_0_/8, 3f_0_/4 with magnitudes in the range of 3–4 kPa. It is postulated that these subharmonics emissions arise from stable chaotically oscillating bubbles, and they are much weaker in magnitude in comparison to transiently collapsing bubble structures attached to the horn tip [40]. The spectrum in the case of MgO de-agglomeration (Fig. 9(b2)) exhibited similar low frequency pressure spectrum with peaks appearing at the fundamental (f_0_), 1st harmonic (2f_0_), ultra-harmonics (5f_0_/4, 4f_0_/3, 7f_0_/4, 25f_0_/12) and subharmonic (2f_0_/3) range. The observed pressure magnitude was found to be highest for 25f_0_/12 with peak amplitude close to 12 kPa followed by 7f_o_/4 with pressure approaching 11 kPa and 2f_0_ with peak pressure of almost 8 kPa. The other low-pressure peaks in the range of 2–7 kPa included 2f_0_/3, f_0_, 4f_0_/3, and 5f_0_/4. These weak subharmonic peaks observed for both silica and magnesia most likely corresponded to large μB clusters that oscillated violently near the agglomerate surface producing de-agglomeration as observed in Fig. 8 (a, b). This is evident in Fig. 8 (a1) at t = 19.08 ms, where a bubbly cluster attached to the silica agglomerate vigorously pulsated with growth and collapse cycle of 0.06 ms reaching maximum size of about 360 μm. This corresponds to a bubble resonance at an oscillating frequency close to 24 kHz, thereby raising a prominent subharmonic peak at 3f_0_/4. Therefore, the bubble cluster dynamics and the overall acoustic emissions can be best described by the range of subharmonic acoustic pressure peaks as previously observed by Tan et al. [41]. The dynamic pressure as a result of shock waves (SW) and microjets arising from these attached bubbles as they undergo splitting and chaotic shape oscillations can reach up to 100 kPa [35]. Unlike intermetallics [26], [27], [28], where the breakage mechanism occurred through the development and propagation of cracks resulting from the SW emissions, the breakage/de-agglomeration of agglomerates was primarily caused by either the gas bubbles present at the surface and within pores of the agglomerate (as also seen with the exfoliation of graphite in [39]) or due to the pressure spikes in the vicinity of the bubble clusters released from the cavitation cloud formed below the vibrating horn producing the pulsations in subharmonics and ultra-harmonics observed in the frequency regime, which are related to the chaotic oscillations and collapse of the bubbly cloud as reported elsewhere [38], [42], [43], [44], [45], [46], [47].

### 3.3 De-agglomeration and dispersion mechanism

**Fig. 10:**
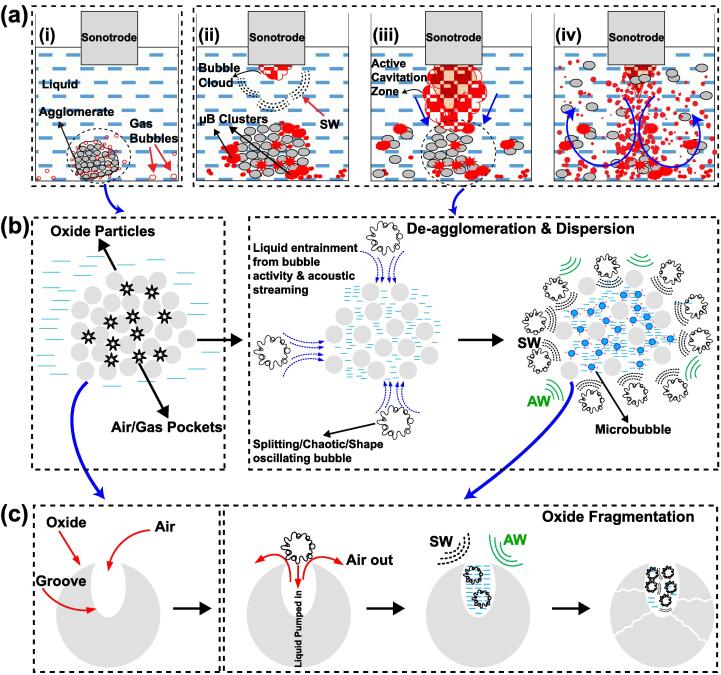
Schematic diagram showing de-agglomeration and fragmentation mechanism of (a, b) an oxide aggregate and (c) single oxide particle, respectively caused by the dynamic pressure effect from oscillating bubbles recognised from the onset of cavitation.

The mechanism of ultrasonic de-agglomeration of oxide agglomerates as observed in Fig. 3, Fig. 4, Fig. 5, Fig. 6, Fig. 7 is depicted in Fig. 10 (a-c). Fig. 10 (a i) shows an oxide agglomerate present at the bottom of a container filled with liquid. There are also gas/vapour bubbles within the pores of the agglomerate and at the surfaces including the container walls. With the inception of cavitation beneath the sonotrode tip, the incoming AW trigger oscillation of these gas bubbles that split and coalesce together to develop μB clusters pulsating near the agglomerate (Fig. 10 (a ii)). As the cavitation cloud becomes bigger and stronger, the μB clusters undergo violent and repetitive rebound and collapse close to the agglomerate, which induces intense shear forces through combined effect of emitted primary SWs [34] and powerful liquid jets [18], [39]. These SWs can further trigger more cavitation cloud collapses generating secondary SWs [26], [48]. It has been previously observed that the overall shock pressure amplitude can range from 1 to 5 MPa at distances up to 3 mm from the horn tip of the same ultrasonic device [26], [39] and is, therefore, sufficient to initiate de-agglomeration by overcoming the interparticle bond strength. This induced pressure can further trigger the gas bubbles present inside and at the surface of the agglomerate to pulsate violently (undergoing splitting and chaotic shape oscillations) promoting de-agglomeration (Fig. 10 (a iii)). De-agglomeration thus also happens from the bulk of agglomerate where the oscillating microbubbles push the oxide particles from within. After the cavitation cloud reaches the agglomerate, the induced acoustic streaming and the developed recirculation vortex disperse the de-agglomerated oxide particles into the bulk liquid and push some of the remaining agglomerates towards the cavitation zone for further fragmentation by high intensity bubble cloud collapses occurring below the sonotrode tip as observed in case of intermetallics [49]. The oscillating μB clusters (acting like drones) also tend to track down the tiny floating agglomerates with size in the range of 300–600 μm by attaching to their surface, promoting their further breakup and subsequent dispersion into fine particles (Fig. 9 (a iv)). The overall rate of de-agglomeration depends on the acoustic pressure generated within the liquid and the amount of stably oscillating and transient μB’s that produce intense stresses upon collapse, sufficient to break the agglomerates to smaller aggregates [50], [51]. Sumitomo et al. [52] reported that the rate of dispersion of the agglomerates was enhanced with low-frequency irradiation. The physical effects of de-agglomeration and dispersion arises from the enhanced mass transport caused by high-energy pressure pulses as a result of μB cluster collapses close to the agglomerate [53]. Therefore, the breakage of agglomerates is not solely induced by SWs as previously reported [52] but also by pulsating and imploding μB clusters through the phenomenon of sono-capillary effect [13], [14], [17]. Fig. 10b shows the representative schematic simplified for better understanding of this phenomenon occurring for a single agglomerate within the liquid. Before sonication, the agglomerate contain many voids or air pockets between the oxide particles, in the form of micro pores and cavities. In reality, however, these individual oxide particles are not regular spheroids but contain grooves on their surface as shown in Fig. 10c. As the cavitation kicks in, splitting bubble clusters undergoing chaotic shape oscillations close to the aggregate, plus the induced acoustic microstreaming force push the surrounding liquid to penetrate inside the agglomerate reaching air pockets and grooves or capillaries. The oscillation of μB clusters as well as the incoming AW/SW near the agglomerate then excites the liquid inside, creating overpressure to nucleate more tiny vigorously oscillating microbubbles that generate large shear forces [54], [55] leading to de-agglomeration and fragmentation of the oxide particles within the bulk liquid. These sequence of events as demonstrated in Fig. 10b is clearly evident with experimental observations made in Fig. 5 (a-f), which highlight the sono-capillary effect occurring on the surface of the aggregate. It can be seen that the oscillating bubble clusters pushes the surrounding liquid into pores of the agglomerate leading to formation of bubbles within the air/gas pockets thereby inflating agglomerate and pushing the oxide particles from inside as observed in Fig. 5 (e-f). Thus, the de-agglomeration of oxides essentially occurs from both direction i.e., ‘chipping-off’ the oxide particles from outer surface of the aggregate [25] and dynamic pressure produced from the chaotically oscillating bubble [35] that pushes the oxide particles from within, like a sandwich mechanism. The majority of agglomerate disintegration is done within few milliseconds of the cavitation inception and the agglomerates that remain unaffected are then tracked down by the floating ‘bubble drones’ making the overall de-agglomeration process very effective.

## 4 Conclusions

In-situ high-speed imaging of cavitation-induced de-agglomeration was performed using high-intensity, low-frequency power ultrasound to fundamentally understand the process mechanism using micron-sized particles of MgO and SiO_2_ agglomerates. Synchronised acoustic pressure measurements were also carried out to characterise the cavitation field in both the time and frequency domain. The following conclusions were drawn from the aforementioned studies:

1. De-agglomeration processes of both oxide aggregates are mechanistically similar and initially occurred through nonlinear acoustic cavitation generated due to ultrasonic excitation of pre-existing gas bubbles upon the introduction of ultrasonic waves within the liquid. The splitting and chaotic shape oscillating bubble clusters induce intense high-energy pressure pulses and the resulting shear stresses eventually chip-off individual oxide particles from the surface of an agglomerate.
2. The increased collapse and rebound of the oscillating gas bubble clusters and the ultrasonic microbubble (μB) clusters near an agglomerate create large overpressure through the ultrasonic-capillary effect facilitating their de-agglomeration also from within. The acoustic streaming then promotes further surface de-agglomeration and dispersion of the loose aggregates. Smaller agglomerates are subsequently captured and separated into individual particles by the floating ‘bubble drones’ within the cavitating medium.
3. Synchronised acoustic pressure measurements reveal that maximum de-agglomeration and dispersion of the oxide clusters occur when the maximum pressure has been reached within the cavitating medium. The generated RMS pressure in the range of 30–50 kPa with maximum pressure surges in the range of 300 kPa was found to be sufficient to induce de-agglomeration.
4. The introduction of ultrasonic waves and cavitation-induced shock waves triggers a strong nonlinear response of the oscillating μB clusters attached to oxide agglomerates raising prominent acoustic pressure peaks in the frequency spectrum, which appear to be associated with the cavitation activity that promotes initial de-agglomeration.

### CRediT authorship contribution statement

**Abhinav Priyadarshi:** Conceptualization, Methodology, Data curation, Software, Validation, Formal analysis, Investigation, Resources, Writing – original draft, Writing – review & editing. **Mohammad Khavari:** Software, Formal analysis, Investigation. **Tungky Subroto:** Resources. **Paul Prentice:** Supervision, Writing – review & editing. **Koulis Pericleous:** Writing – review & editing, Supervision, Funding acquisition. **Dmitry Eskin:** Writing – review & editing, Supervision, Funding acquisition. **John Durodola:** Supervision. **Iakovos Tzanakis:** Conceptualization, Methodology, Resources, Writing – review & editing, Supervision, Funding acquisition.

## Declaration of Competing Interest

The authors declare that they have no known competing financial interests or personal relationships that could have appeared to influence the work reported in this paper.
